# The Major Intrinsic Protein Family and Their Function Under Salt-Stress in Peanut

**DOI:** 10.3389/fgene.2021.639585

**Published:** 2021-02-24

**Authors:** Yan Han, Rongchong Li, Yiyang Liu, Shoujin Fan, Shubo Wan, Xuejie Zhang, Guowei Li

**Affiliations:** ^1^Shandong Provincial Key Laboratory of Plant Stress, College of Life Sciences, Shandong Normal University, Ji’nan, China; ^2^Key Laboratory of Crop Genetic Improvement & Ecology and Physiology, Bio-technology Research Center, Shandong Academy of Agricultural Sciences, Ji’nan, China

**Keywords:** aquaporin, peanut, salt stress, seed germination, gene family

## Abstract

Peanut (*Arachis hypogaea*) is an important oil crop cultivated across the world. Abiotic stresses are the major constraint factors that defect its yield, especially in the rainfed peanut cultivation areas. Aquaporins are proteins that form a large family of more than 30 members in higher plants and play key roles in plant water balance under abiotic stress conditions. To comprehensively understand the functions of aquaporins in peanut, we identified their family genome-wide and characterized the phylogenetics, gene structure, and the conserved motif of the selective filter. In total, 64 aquaporin isoforms were identified, the NIPs were firstly categorized into NIP1s and NIP2s groups based on the phylogenetic analysis and the selective filter structure classification system. Further, we analyzed the gene expression pattern under the salt-stress conditions and found that a TIP3 member is strongly induced by salt stress, which in turn contributed to improved seed germination under salt stress when expressed in Arabidopsis. Our study thus provides comprehensive profiles on the MIP superfamily and their expression and function under salt-stress conditions. We believe that our findings will facilitate the better understanding of the roles of aquaporins in peanuts under salt salt-stress conditions.

## Highlights

- We identified 64 complete aquaporin isoforms and firstly suggested to categorize the NIP subfamily in plants into NIP1s and NIP2s groups based on the phylogenetic analysis and the selective filter structure classification system.
- Further, we identified a salt stress responding aquaporin isoform AhTIP3;1 and experimentally confirmed that it contributed to improved seed germination under salt stress when expressed in *Arabidopsis.*

## Introduction

Water balance is a vital factor that contributes to plant growth and development. Soil, terrestrial plants, and the atmosphere make a continuum for water transmission. In plants, aquaporins play key roles in water uptake by the roots and its distribution in tissues. The term “aquaporin” is presently applied in a broad sense when referring to all plant major intrinsic proteins (MIPs), including not only the isoforms that function as strict water channels, but also those that function as small neutral molecule transporters (Maurel et al., 2008). It has been reported that aquaporins, which are localized in the plasma membrane, endoplasmic reticulum, vacuoles, and plastids, are involved in various plant physiological aspects from seed germination, seedling development and seed maturity, and response to biotic and abiotic stress responses (Li et al., 2014).

According to their homology and localization on the membrane, plant aquaporin homologs can be classified into four common subfamilies that are present in most species, including plasma membrane intrinsic proteins (PIPs; primarily localize on the plasma membrane); tonoplast intrinsic proteins (TIPs; target to the vacuolar membrane); NOD26-like intrinsic proteins (NIPs; localize to the plasma membrane or the endoplasmic reticulum), and small basic intrinsic proteins (SIPs). The three specific subfamilies include the uncategorized X intrinsic proteins (XIPs) discovered in protozoa, fungi, and partial higher plants; GlpF-like intrinsic proteins (GIPs), and hybrid intrinsic proteins (HIPs) that are present exclusively in moss (Maurel et al., 2015). During the course of evolution, the GIPs and HIPs were lost in dicots and, further, the XIPs were lost in monocots (Danielson and Johanson, 2008). Past studies have shown the presence of a great diversity in aquaporin localization, expression, function, and regulation with >30 isoforms in higher plants, and its family size has almost doubled in the polyploidy species (Maurel et al., 2015). Aquaporins play a basic role in the water transport regulation, although some isoforms can also transport additional or special substrates including H_2_O_2_, CO_2_, NH_3_, urea, glycerol, boron, silicon, and selenium. For instance, the root hydraulic conductivity in *Arabidopsis* decreased by 20% and 40% in *pip1;2* and *pip2;1pip2;2*, respectively (Peret et al., 2012). NtAQP1 and AtPIP1;2 play dual roles in water and CO_2_ transport (Siefritz et al., 2001; Uehlein et al., 2003; Postaire et al., 2010; Heckwolf et al., 2011). AtNIP5;1 acts as a boron transport channel in the regulation of plant development under boron-limited conditions (Takano et al., 2006). For the legumes, symbiotic root nodules are developed to fix nitrogen (N_2_), and nodulin-26 (*GmNOD26*) from soybean was the first identified aquaporin (Fortin et al., 1987). In addition, NIP and TIP isoforms play roles in nodule formation and NH_4_^+^/NH_3_ translocation (Guenther and Roberts, 2000; Hwang et al., 2010; Gavrin et al., 2014).

Peanut (*Arachis hypogaea* L.), which is one of the major legume crops worldwide, is cultivated in over 80 countries ranging from the warm temperate to the tropical regions, with the maximum production in Asia, Africa, and Americas (Wright and Rao, 1994). Salinity can defect seed germination, seedling growth and inhibit photosynthesis and Ca^2+^, K^+^, and Mg^2+^ deficiency in peanut (Cui et al., 2018). External potassium application can improve salinity tolerance and it was reported that calcium has interactive effects with sodium on the accumulation of proline and glycinebetaine in peanut (Girija et al., 2002; Chakraborty et al., 2016). Calcium might also play a role in water balance during salt stress as that it have been proved to regulate water transport activity of aquaporins in Arabidopsis (Verdoucq et al., 2008). However, the roles of aquaporins in water balance and other physiological processes are unclear, necessitating the characterization of the MIPs to facilitate our understanding of their roles in peanut.

## Materials and Methods

### Identification of MIPs in *A. hypogaea*

To identify the potential members of the aquaporin family, the *A. hypogea* genome sequences were retrieved from the PeanutBase^1^ by using Pfam ID PF00230 for major intrinsic proteins^2^ (Finn et al., 2016). The sequences with two NPA motif, six transmembrane domains, and five loops were considered as complete MIPs. The gene location on the chromosomes and the length of gene, transcript, coding sequence, and protein are shown in Supplementary Table 1. The isoelectric point (*p*I) calculation tool from ExPASy^3^ was used to predict the *p*I (Stothard, 2000).

### Phylogenetic Analysis, Gene Structure Analysis, and Conserved Motif Prediction

The phylogenetic tree was constructed using MEGA with full-length protein sequences of aquaporins from peanut, *Arabidopsis*, and soybean (*Glycine max*) (Kumar et al., 2008). The neighbor-joining (NJ) method was adopted with the application of 1000 bootstrap replicates. The exon–intron characteristics of the MIPs were exhibited using the Gene Structure Display Server (GSDS 2.0) by comparison with the full-length predicted gene coding sequences (Hu et al., 2015). The conserved motifs were defined using the MEME.^4^

### The Expression Analysis of Aquaporins in *A. hypogaea*

Our previous RNA-seq data from the Genbank BioProject PRJNA398720 was employed to characterize the tissue-specific expression and their response to salt stress of aquaporin family. The transcriptome assembly and expression value were estimated as described elsewhere (Cui et al., 2018). The gene relative expression was normalized to the Reads Per Kilobase of transcript per Million mapped reads (RPKM), and the aquaporin genes identified were used for heat map by using the TIGR Multi Experiment Viewer (MeV).^5^ Hierarchical clustering with average linkage method was performed to cluster the samples. The gene expression in various tissues were investigated by quantitative-polymerase chain reaction (qPCR), and the samples were prepared as previously described (Clevenger et al., 2016). The tissues for gene expression analysis were collected, including radicles, shoots and roots from 20-day-old seedlings, flowers, and aerial and subterranean pegs and fruits in five stages, as described elsewhere (Liu et al., 2019).

### Transformation and Seed Germination Assay Under Salt Tolerance

Total RNA was extracted from the cultivar cv. Tifrunner with TRNzol reagent, and the cDNA was synthesized with the oligo (dT)_18_ primer and Rever-Tra Ace M-MLV RTase in a total reaction volume of 20 μL. The open reading frame (ORF) of *AhTIP3;1* was amplified by PCR with the following primers: FP-ATGGCTACTAGAAGATATGCTTTTG, RP-CTAGTAATC TTCAGGAGCCAAC. The PCR fragment was confirmed by sequencing. The full-length *AhTIP3;1* was inserted in PHB vector and the transformation of Col-0 by using Agrobacterium tumefaciens strain GV3101. Transgenic lines were screened on MS plates containing 50 mg/mL hygromycin as previously described (Qin et al., 2019). qPCR was employed to investigate the relative expression in the transgenic lines with primers (FP: ACTCATCAACCGTTGGCTCCTG; RP: ACAAGACACAAAG AGAAACCCCAC). *AtACT7* (AT5G09810; FP: CTGATGT CGCCGTGCTCTTGG; RP: CTGTTGAGGTTGGTGTAGGTA GG) and *TUA5* (Arahy.56W2G2; FP: TCCATGAAACAAC TTACAACTCCATCA; RP: CATCGTACTCACTCTTTGAAAT CCACA) were employed as the reference genes in peanut and *Arabidopsis*, respectively.

### Water Permeability Analysis

*AhTIP3;1* was amplified from the PHB-AhTIP3;1 by PCR with ORF cloning primers plus *Bgl*II recognition sequence AGATCT, and cloned into the *Bgl*II site of pXbG-ev1 vector. Water permeability analysis was performed as previously described (Li G. W. et al., 2008). In briefly, the capped *AhTIP3;1* RNA transcript was synthesized *in vitro* using a kit from the mMESSAGE mMACHINE. The good quality oocyte was selected for injection and each was injected with 23 ng of cRNA or the same volume of sterile water as control. Fully grown oocytes (stage V and VI) were isolated from *Xenopus laevis* and incubated on Barth’s solution as previously described (Prado et al., 2019). The oocyte swelling assay was performed after incubation for 3 days at 18°C in Barth’s buffer, and the *P*_*f*_ was calculated based on the initial oocyte swelling rate.

### Plant Culture and Treatments

Peanut seedlings were grown in the greenhouse for mRNA extraction and transcriptomic analysis. Col-0 for genetic transformation was grown in a growth chamber with 16-h light (200 μmole photons m^–2^s^–1^) and 8-h dark photocycle at 20°C (Qin et al., 2019). For the germination assay under salt stress, the seeds of wild type and transgenic lines were spread on salt-free 1/2MS medium or supplemented with 100 or 150 mM NaCl in the growth chamber, and the germination rate was counted daily for 7 days after maintaining at 4°C for 72 h (Vander et al., 2006).

## Results

### Genome-Wide Identification of MIPs in *A. hypogaea*

Homology-based research was conducted for the *A. hypogaea* genome of the cultivar cv. Tifrunner (Bertioli et al., 2019), whereby 64 aquaporins were identified based on the typical characters of six transmembrane domains and conserved NPA motif in the loops B and E, respectively. The aquaporins were distributed on 16 chromosomes, with half on the A (Chr1-10) and half on the B (Chr11-20) genome. There was a total of 29 pairs of corresponding homologs on the A and B genome due to the genome duplication. Most of the isoforms (14 isoforms including 6 corresponding pairs) were distributed on Chr3 and Chr13, but no isoforms were distributed on Chr1, Chr2, Chr11, and Chr12. The gene size varied from 1,807 bp (*AhNIP1;3*) to 12,537 bp (*AhNIP7;1*), and the proteins with 216 (AhNIP3;1) to 371 (AhXIP2;2) amino acids. The *p*I of most aquaporins was ≥7.0; however, most of the isoforms from the TIP subfamily localized on the tonoplast membrane had lower *p*I values, except for AhTIP1;3 and AhTIP1;5 (Supplementary Table 1).

### Phylogenetic and Gene Structure Analysis of Aquaporins

**FIGURE 1:**
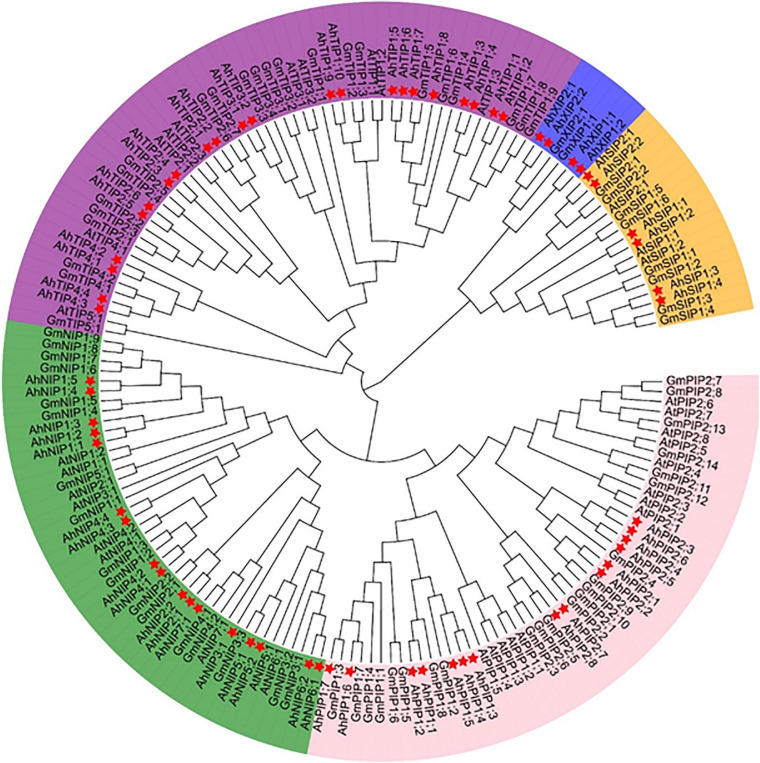
Phylogenetic tree of aquaporins identified from *Arachis hypogaea* along with the isoforms from *Arabidopsis thaliana* and *Glycine max*. The 64 aquaporins are categorized into five subfamilies: PIPs, TIPs, NIPs, SIPs, and XIPs. The details of the aquaporins from *Arachis hypogaea* are available in Supplementary Table 1, and the isoform sequence information of *A. thaliana* and *G. max* is with reference to the report by Shivaraj et al. (2019). The genes from *A. hypogea*, *G. max*, and *A. thaliana* are preceded by the prefixes Ah, At, and Gm, respectively. The isoforms from *A. hypogea* are indicated with asterisks.

The MIP family could be divided into seven subfamilies (Maurel et al., 2015) and we investigated the evolutionary relationship of aquaporins from *A. hypogaea* compared with the homologs from *Arabidopsis* and soybean. The aquaporin gene number in *A. hypogaea* is almost double compared with that in *Arabidopsis* (35 isoforms), and it is comparable to that in soybean (72 isoforms) (Deshmukh et al., 2016). The phylogenetic analysis indicated that the aquaporins in *A. hypogaea* could be classified into the following five subfamilies: 18 PIPs, 22 TIPs, 14 NIPs, 6 SIPs, and 4 XIPs, but without homologs from the GIP or HIP subfamilies (Figure 1). The PIP1s from peanut and soybean were mixed in two clades, but those from *Arabidopsis* were separated. All of the eight PIP2s from *Arabidopsis* together with five PIP2s from soybean were clustered in one clade, and the eight PIP2s from peanut with the other 10 PIP2s from soybean were clustered in two clades. Totally, the TIPs from the three species were classified into five subgroups, and the TIP1 subgroup included the greatest number of members with 10 isoforms. However, no corresponding TIP5 isoforms was identified in *A. hypogaea*. All the NIPs from *Arabidopsis*, soybean and peanut were clearly divided into two groups, which is completely consistent with the classification based on the selective filter (Supplementary Table 2).

### Characterization of NPA Motif and Amino Acid Residues of the Selective Filter

According to the NPA (Asn-Pro-Ala) motif and the selective filter [aromatic/arginine (ar/R)] theory, the NPA motifs in the loop B (LB) and loop E (LE) and the four amino acid residues in helix 2 (H2), helix 5 (H5), LE_1_, and LE_2_ play key roles in transport selectivity and activity (Wallace and Roberts, 2004). The PIP subfamily possesses the mostly conserved sequences in the NPA motif and a uniform ar/R signature, which is conserved in most plant PIPs identified until date. For the TIP subfamily, the groups I and II are consistent with those reported in *Arabidopsis*, except that the NPA in loop B changed to NPV in group IA (AhTIP1;1 and AhTIP1;2). The group III (TIP5s) was missed in *A. hypogaea*, but a novel group III was identified with AhTIP4;1 and AhTIP4;2. The latter 3 ar/R residues in the novel group III was consistent with that in group II, but the His (H2) in group II was substituted with Ser (H2). More variation was noted in the residues of the selective filter in the SIP subfamily when compared with that in *Arabidopsis*. The XIPs were divided into two groups based on the residues of the selective filter (Supplementary Table 2).

**FIGURE 2:**
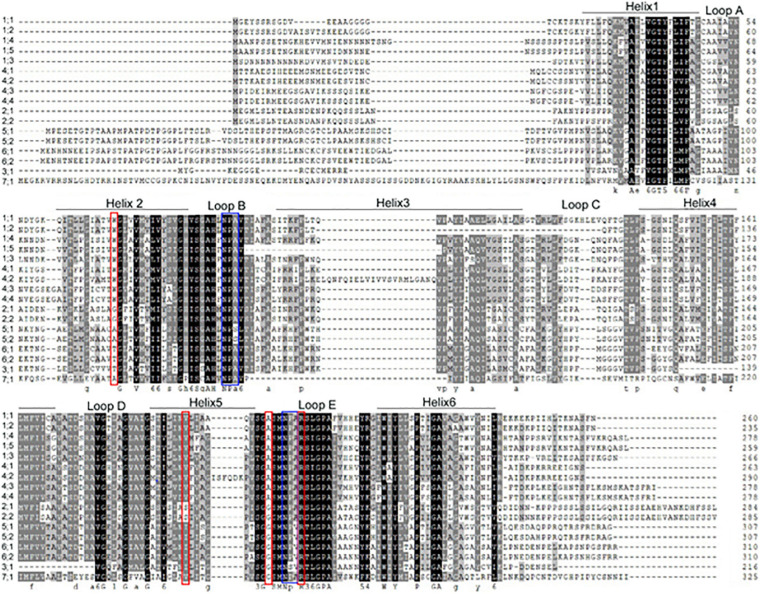
Alignment of the deduced amino acid sequences of all NIP isoforms in peanut. Amino acids that are conserved in these sequences are highlighted in gray. The 6 transmembrane α-helices (Helix1-6) are indicated with a bar and the connecting loops are labeled Loop A–E. The conserved NPA motifs are located in loops B and E are boxed in blue. The four selective filter residues are boxed in red.

There are more diversity in the sequence and function of NIP isoforms, which was further divided into seven subgroups in several species according to the phylogenetics analysis. However, it was classified into two groups according to the ar/R signature in Arabidopsis (Wallace and Roberts, 2004). In peanut, the NIP subfamily isoforms can be divided into two groups according to the ar/R signature (Figure 2 and Supplementary Table 2). The amino acid residues of the ar/R signature (WVAR) in group I (AhNIP1;1-1;5, AhNIP4;1-4;4) is consistent to that in the corresponding groups I in *Arabidopsis*. The NIP isoforms in group II can be divided into IIA and IIB subgroups. The group IIA includes three isoforms (AhNIP5;1-5;2, AhNIP7;1) possessing selective filter AI(V)GR consistent with that in Arabidopsis, and three isoforms (AhNIP3;1, AhNIP6;1-6;2) in peanut with selective filter TIGR. The group IIB isoforms (AhNIP2;1-2:2) have same selective filter GSGR with silicic acid transporter OsLsi1 (OsNIP2;1) in rice, which implies that AhNIP2;1 and AhNIP2;2 may play a role in silicic acid transport (Mitani-Ueno et al., 2011).

### Aquaporin Expression Profiling in Response to Salt Stress

**FIGURE 3:**
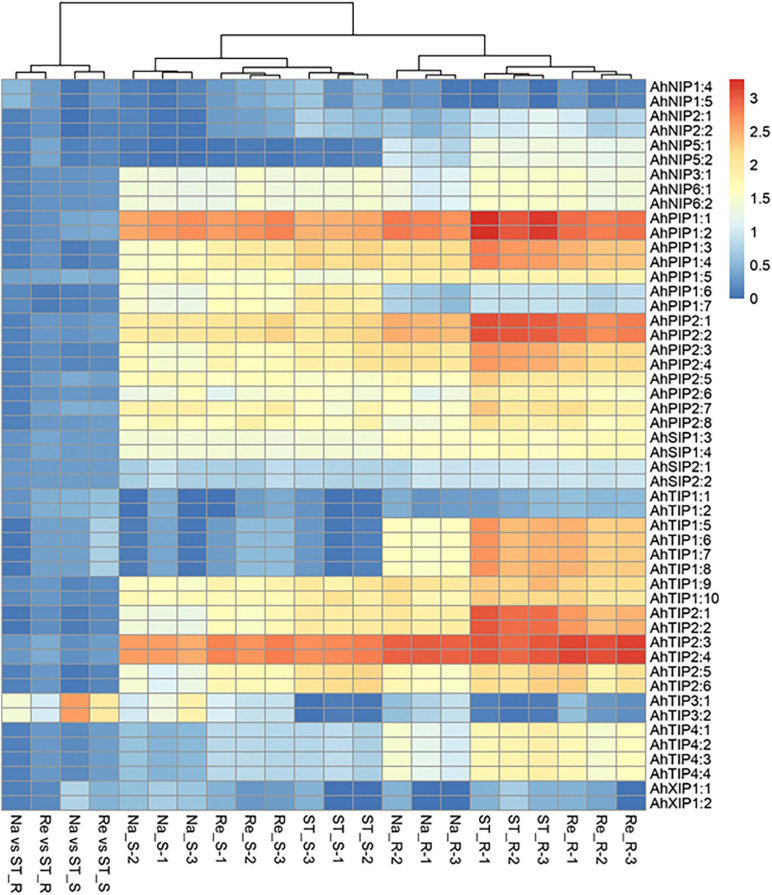
The aquaporin expression and response to salt stress in an *Arachis hypogea* cultivar Luhua14 using RNA-seq data (PRJNA398720 BioProject). The expression of aquaporins was normalized by reads per kilobase of transcript per million mapped reads (RPKM) under the standard conditions (ST), salt stress (Na), and recovery (Re) in the shoots (_S) and roots (_R) with 3 replicates. The relative expression in salt stress (Na vs ST) and recovery (Re vs ST) conditions were calculated from the Na and Re against ST, respectively.

**FIGURE 4:**
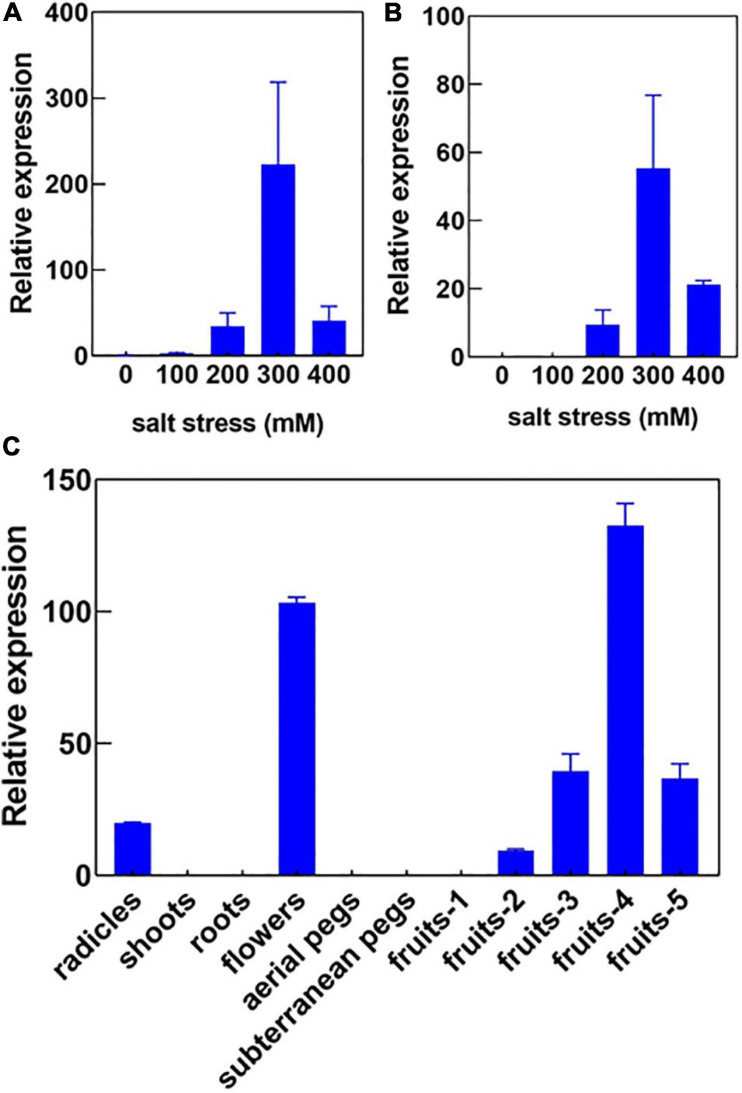
The expression pattern of *AhTIP3;1* in response to salt stress and in various tissues. The relative expression of *AhTIP3;1* in response to 100, 200, 300 and 400 mM NaCl in the roots **(A)** and shoots **(B)** by quantitative PCR. The relative expression of *AhTIP3;1* in the radicles, shoots, roots, flowers, aerial pegs, subterranean pegs, and fruits under different stages by quantitative PCR **(C)**.

In our previous study, we had investigated the effect of salt stress on a peanut cultivar Luhua14 (Cui et al., 2018). By using the RNA-seq data, we identified 50 aquaporins including 15 PIPs, 20 TIPs, 9 NIPs, 4 SIPs, and 2 XIPs in the shoots or roots under the standard or salt stress conditions. Data shows that eight aquaporins, including four PIPs and four TIPs (*AhPIP1;1*, *AhPIP1;2*, *AhPIP2;1*, *AhPIP2;2*, *AhTIP2;1*, *AhTIP2;2*, *AhTIP2;3*, and *AhTIP2;4*) were highly expressed both in the shoots and in the roots under the standard conditions. The expression of most aquaporins identified was repressed; however, *AhTIP3;1* and *AhTIP3;2* were induced by salt stress (Figure 3). The expression pattern of *AhTIP3;1* was further confirmed both in the roots and in the shoots under salt stress conditions (200, 300, and 400 mM NaCl) by qPCR (Figures 4A,B).

### The Expression and Functional Analysis of AhTIP3:1

Usually TIP3s are considered as seed-specific expression proteins and used as markers for vacuoles. Here, we investigated the expression of *AhTIP3;1* in various tissues, including the radicles, shoots, roots, aerial and subterranean pegs, and fruits at 5 different stages. Our data showed that *AhTIP3;1* was highly expressed in the flowers and fruits at stage 3–5, relatively expressed in the radicles, and scarcely expressed in the shoots or roots (Figure 4C); these findings were consistent with the RNA-seq data (Figure 3).

**FIGURE 5:**
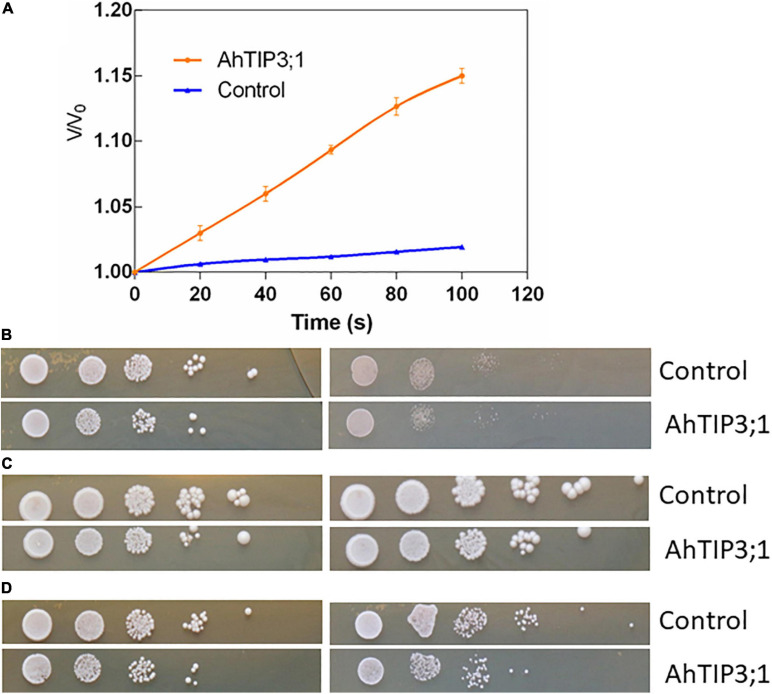
The function assay of AhTIP3;1. The time course of osmotic swelling of individual Xenopus oocytes. The oocytes were injected with complementary RNA of AhTIP3;1. or water as control. After incubation in Barth’s solution for 3 days, oocytes were exposed to diluted Barth’s solution from *t* = 0 and the oocyte swelling rate V/V_0_ was plotted against time. The *P*_*f*_ values were calculated from the initial rate of oocyte swelling **(A)**. Yeast cells of W303 harboring the AhTIP3;1 expressing construct (AhTIP3;1), and yeast cells with the vector pYES2 only (control) were dropped on YPD plate with 0.5 M NaCl and 1.0 M **(B)**, 1 and 2 mM NH_3_ **(C)** and 20 and 40 mM Boron **(D)**. Cell density was adjusted to OD-600 at 1.0 and serial dilutions were made at each step and 10 μL each dilution was spotted at each point. The plates were incubation at 30°C for 48 h before photographs were taken.

Xenopus oocyte expression system is effective method to test the MIP member’s transport activity. The water permeability assay showed that AhTIP3;1 has a higher water transport activity when expressed in oocytes. The osmotic water permeability (*P*_*f*_) was calculated from the rate of oocyte cell volume variation in high water potential solution. The cell volume increased by 18.33% after 110 s when expressed with AhTIP3;1. The corresponding *P*_*f*_ value is approximately 0.503 × 10^–2^cm/s. However, the volume of the control cell injected with water enhanced by 2%–3%, which indicates a very low water permeability (Figure 5A).

Yeast expression system has been employed to investigate the functions of MIP isoforms. The data showed that AhTIP3;1 expressed yeast strains were more sensitive to salt stress compared to the control strains transformed with empty vector (Figure 5B). The growth of the AhTIP3;1 expressed yeast strains was weaker on the YPD media with NH_3_ or Boron compared to that transformed with empty vector, which suggested that AhTIP3;1 may have somehow NH_3_ and Boron transport activity (Figures 5C,D).

**FIGURE 6:**
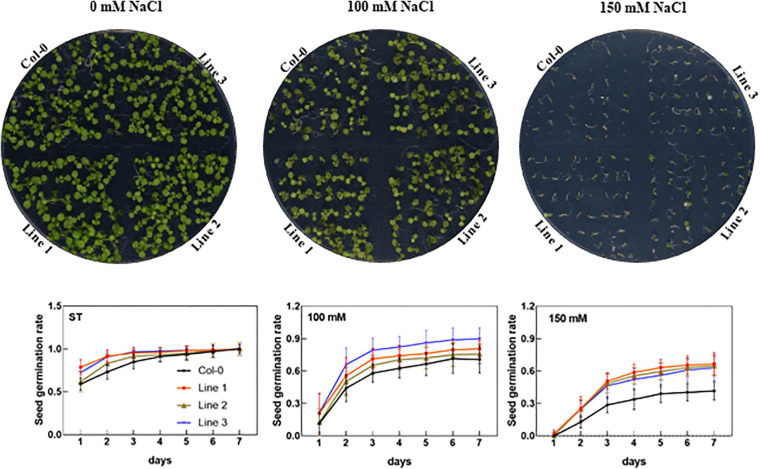
The effect of salt stress on the seed germination of AhTIP3;1 overexpressed lines (line 1–3). The images of the AhTIP3;1 overexpressed lines and Col-0 growth on the MS/2 media after 7 days (upper lane). The seed germination rate under salt stress (100 and 150 mM NaCl) and standard conditions (ST) (lower lane). The experiments were done in triplicate and all the data was presented as mean ± standard error (SE).

To investigate the physiological functions, we further cloned *AhTIP3;1* and transformed into *Arabidopsis*. Three highly over-expressed lines of AhTIP3;1 (Lines 1–3) were used to investigate seed germination under the salt-stress conditions. Our data showed that the seed germination did not change in the over-expressed lines compared to that in the wild type under the standard conditions. However, the seed germination of the *AhTIP3;1* over-expressed lines was higher when compared to that of Col-0 under 100 and 150 mM NaCl treatments (Figure 6).

## Discussion

The aquaporin evolved from prokaryota to eukaryote during the evolution (Finn and Cerda, 2015). As a result, the gene number of aquaporin gradually increased during the evolution, and it finally emerged with a gene diversity in higher plants that is much more complex than that of bacteria, moss. The GIPs in the plants may have originated via horizontal gene transfer from an ancestral bacterial gene, but it was lost in higher plants during evolution (Gustavsson et al., 2005). PIPs, XIPs, and SIPs in higher plants were likely inherited from the algal ancestors, while HIPs and TIPs were probably derived from a PIP ancestor (Maurel et al., 2015). The origin of plant NIPs arose from bacterial AqpN (Finn and Cerda, 2015). The differences in the numbers of aquaporin paralogs are believed to be associated with tandem duplication and the degree of polyploidy among the majority of flowering plants. In wild diploid peanut, there are approximately 30 aquaporin isoforms (Shivaraj et al., 2019), and in the tetraploid peanut, the isoform number doubles, with most isoforms in A genome having a corresponding homolog in B genome, which is linked to gene duplication during evolution (Figure 1 and Supplementary Table 1). Despite the greater diversity in homology, as in the PIP subfamily in plants, the entire NIP subfamily from *Arabidopsis*, soybean, and peanut are clearly divided into two clades, which is completely consistent with the classification system based on the selective filter structure (Wallace and Roberts, 2004). Based on this analysis, we suggest the division of NIPs into NIP1s and NIP2s groups in the future as well as further investigation to detect possible common transport functions within the isoforms among the two clades.

Tissue dehydration is a common effect of several abiotic stresses, including drought and salt stress, in which the water balance between the root water uptake and leaf transpiration is disturbed (Aroca et al., 2012). Past evidences have shown that aquaporins play a positive or negative role in plant water balance under abiotic stress conditions, although the exact mechanisms remain unclear. For instance, SlTIP2;2, a stress-induced aquaporin in tomato, can enhance transpiration and modify the leaf water potential and enhance the fruit yield under drought conditions (Sade et al., 2009). Moreover, it can further regulate the Na^+^ and K^+^ balance under salt-stress condition when expressed in *Arabidopsis* (Xin et al., 2014). *A. hypogaea* is considered as a moderately salt-tolerant species, and several aquaporins vary in response to salt stress (Figure 3). These facts imply that aquaporins may involve in water balance in the shoots and roots under salt-stress conditions.

Several lines of data support that aquaporins play a key role in seed germination (Maurel et al., 2015). The expression of PIP1s, PIP2s, TIP1, and TIP3 were accumulated at the translational level (Vander et al., 2006; Liu et al., 2007, 2013). The seed size and salt tolerance reportedly increases in the *Arabidopsis* and soybean lines overexpressed with PgTIP1 from ginseng (Lin et al., 2007; Peng et al., 2007; An et al., 2017). In rice, the accumulation of OsPIP1;3 in the seeds and the seed germination rate were positively related to its expression level under the control of NO signaling (Liu et al., 2007). TIPs were widely used as markers for vacuolar compartments in the higher plants, and TIP3s are usually considered as seed-specific expressed isoforms and employed as protein storage vacuole markers (Jauh et al., 1999). TIP3s were accumulated during seed maturation and degraded with seed germination, which may be related to the deposition of storage proteins, oligosaccharides, and phytins in protein storage vacuoles (Maurel et al., 1997; Li G. et al., 2008). AhTIP3s in *A. hypogaea* belonging to the Group I TIPs based on the selective filter structure was also found to be highly accumulated during seed maturation, which indicates that AhTIP3s play roles in seed physiology with seed maturation (Figure 6).

## Data Availability Statement

The original contributions presented in the study are included in the article/Supplementary Material, further inquiries can be directed to the corresponding author/s.

## Ethics Statement

The animal study was reviewed and approved by the Ethics committee of Bio-tech Research Center of SAAS. Written informed consent was obtained from the owners for the participation of their animals in this study.

## Author Contributions

SF, SW, and GL planned and designed the research. YH, YL, and XZ performed the experiments. RL and YH collected the data and conducted the analysis. XZ and GL wrote the manuscript. All authors contributed to the article and approved the submitted version.

## Conflict of Interest

The authors declare that the research was conducted in the absence of any commercial or financial relationships that could be construed as a potential conflict of interest.

## Footnotes

1 https://peanutbase.org/

2 http://pfam.xfam.org/search#tabview=tab0

3 http://web.expasy.org/compute_pi/

4 http://meme-suite.org/tools/meme

5 http://mev.tm4.org
